# Adiponectin Interacts *In-Vitro* With Cementoblasts Influencing Cell Migration, Proliferation and Cementogenesis Partly Through the MAPK Signaling Pathway

**DOI:** 10.3389/fphar.2020.585346

**Published:** 2020-12-22

**Authors:** Jiawen Yong, Julia von Bremen, Gisela Ruiz-Heiland, Sabine Ruf

**Affiliations:** Department of Orthodontics, Faculty of Medicine, Justus Liebig University Giessen, Giessen, Germany

**Keywords:** adiponectin, cementoblasts, migration, proliferation, cementogenesis, MAPK

## Abstract

Current clinical evidences suggest that circulating Adipokines such as Adiponectin can influence the ratio of orthodontic tooth movement. We aimed to investigate the effect that Adiponectin has on cementoblasts (OCCM-30) and on the intracellular signaling molecules of Mitogen-activated protein kinase (MAPK). We demonstrated that OCCM-30 cells express AdipoR1 and AdipoR2. Alizarin Red S staining revealed that Adiponectin increases mineralized nodule formation and quantitative AP activity in a dose-dependent manner. Adiponectin up-regulates the mRNA levels of *AP*, *BSP*, *OCN*, *OPG*, *Runx-2* as well as *F-Spondin*. Adiponectin also increases the migration and proliferation of OCCM-30 cells. Moreover, Adiponectin induces a transient activation of JNK, P38, ERK1/2 and promotes the phosphorylation of STAT1 and STAT3. The activation of Adiponectin-mediated migration and proliferation was attenuated after pharmacological inhibition of P38, ERK1/2 and JNK in different degrees, whereas mineralization was facilitated by MAPK inhibition in varying degrees. Based on our results, Adiponectin favorably affect OCCM-30 cell migration, proliferation as well as cementogenesis. One of the underlying mechanisms is the activation of MAPK signaling pathway.

## Introduction

The periodontium consists of the gingiva, periodontal ligament, root cementum and alveolar bone (Park et al., 2017). Cementum, a heterogeneous mineralized layer covering the entire root dentin surface, anchors fibrous connective tissues on tooth-root surfaces (Caverzasio and Manen, 2007). As a special mineralized tissue, cementum has a similar composition to bone, consisting of approximately 61% mineralized as well as 27% organic matrix and 12% water (Nanci and Bosshardt, 2006).

Cementoblasts are highly differentiated mesenchymal cells of the periodontal ligament (PDL) with the capacity to build up cementum (Arzate et al., 2015). The cementum matrix is composed of collagenous proteins and non-collagenous proteins such as Bone Sialoprotein (BSP), Osteopontin (OPN), Osteocalcin (OCN), Osteoprotegerin (OPG), Fibronectin, Osteonectin and several growth factors (Saygin et al., 2000).

It has been demonstrated that Adipocytokines, which are mainly secreted by adipose tissue, can influence bone metabolism (Funahashi et al., 1999). Adiponectin, which is produced by adipocytes but also by salivary gland epithelial cells, has been shown to modulate metabolic and immune functions of salivary gland epithelial cell (Katsiougiannis et al., 2006).

Two Adiponectin receptor (AdipoRs) subtypes (Adiponectin receptor 1 and 2 (AdipoR1 and AdipoR2)), expressed on various tissues and cells such as human gingival fibroblasts and PDL cells (Yamauchi et al., 2003a) mediate the biological effects of Adiponectin. Yamaguchi et al. (2012) showed that the AdipoRs were ubiquitously expressed in the oral tissues of mice such as gingiva, tongue, buccal mucosa and labial mucosa, as well as in RAW24 cells (Yamauchi et al., 2012). Both AdipoRs exert similar effects but have individual signaling preferences. Whereas phosphorylation of Extracellular Regulated Kinase 1/2 (ERK1/2) may depend on both receptors, AdipoR1 is more prominent in AMP-activated protein kinase (AMPK) phosphorylation, and AdipoR2 is involved in PPAR-α activation in muscle cells (Buechler et al., 2010). AdipoR1 and AdipoR2 have been identified in the plasma membrane (Yamauchi et al., 2003b) as well as in the cytoplasm (cytoplasmic puncta) (Ding et al., 2009).

*In-vivo*, Adiponectin secretion is regulated by the degree of obesity (Carbone et al., 2012). Its secretion is inversely proportional to the amount of adipose tissue (Swarbrick and Havel, 2008; Benedix et al., 2011; de Carvalho et al., 2017). It has been shown that patients with type 2 diabetes and obesity have lower serum Adiponectin levels than normal weight individuals (Cao et al., 2015). Furthermore, clinical studies have analyzed the relationship between the body mass index (BMI) and salivary Adiponectin levels (Mamali et al., 2012; Nigro et al., 2015) and found a trend toward slightly higher expression of salivary Adiponectin in obese patients than in controls, however they could not show a statistic significance between groups, whereas they previously reported that Adiponectin serum levels are reduced in obese patients compared to healthy controls. Also, patients with severe periodontitis had lower Adiponectin- and AdipoRs-levels than healthy subjects (Saito et al., 2008; Yamaguchi et al., 2010).

Recent studies revealed that local submucosal injection of Adiponectin prevented experimental orthodontic tooth movement in rats (Haugen et al., 2017). The data suggested that Adiponectin is involved in the homeostasis of periodontal tissues and thus might influence orthodontic treatment.

It has been demonstrated that Adiponectin and its receptors are also expressed in bone marrow-derived osteoblasts and adipocytes (Seo et al., 2004), suggesting that Adiponectin may play a role in bone metabolism (Chen et al., 2013). Considering the multifunctional role of Adiponectin, it seems possible that it may have functional characteristics in cementoblasts similar to that in other mineralized tissue-forming cells such as osteoblasts (Seo et al., 2004).

Since fat mass may impact peripheral bone formation, it may be one of the critical factors for orthodontic tooth movement rate and orthodontically-induced tooth root resorption. Adipocyte-derived hormones such as Adiponectin may contribute to this relationship. To date, the physiological effect of Adiponectin on cementoblasts has not been elucidated. The decreased levels of Adiponectin, as found in obese individuals, might be a critical pathomechanistic link. Therefore, the present study was undertaken to investigate the effect of Adiponectin on OCCM-30 cells *in vitro*.

## Materials and Methods

### Cell Culture and Reagents

The OCCM-30 cementoblast cell line was kindly provided by Prof. M. Somerman (NIH, NIDCR, Bethesda, MA) and cultured as previously described (D’Errico et al., 2000). Briefly, cells were maintained in α-MEM (11095-080, Gibco) containing 10% Fetal Bovine Serum (FBS) (10270-106, Gibco) and 1% Penicillin/Streptomycin (15140-122, Gibco) and incubated in a humidified atmosphere of 5% CO_2_ at 37°C. The cells were seeded into 6-well plates (657160, Greiner Bio-one) at a density of 3 × 10^4^ cells/well until confluence. The cells used were between passages 3 and 7. To induce cementogenesis, the cell culture medium was supplemented with 10 mM β-Glycerophosphate (#35675, Calbiochem) and 50 μg/ml Ascorbic Acid (6288.1, Roth).

Cells were stimulated using different concentrations of mouse Adiponectin/Acrp30/ADIPOQ protein (His Tag) from Sino Biological Inc. (Cat. No: 50636-M08H). Purity > 95% (Determined by SDS-PAGE). Endotoxin < 1.0 EU/μg (Determined by the LAL method). Protein construction: The DNA sequence encoding mouse ADIPOQ (NP_033735.3) (Met 1-Asn 247) was expressed with a C-terminal polyhistidine tag. Expression Host: HEK 293 cells. Formulation: Lyophilized from sterile PBS, pH 7.4, 5% Trehalose, 5% Mannitol, 0.01% Tween-80. The protein was reconstituted following manufacturer indications to a stock solution of 0.25 mg/ml in sterile water and stored at −20°C. The MAPK inhibitor for P38 (SB203580) (#tlrl-sb20, InvivoGen), the ERK1/2 inhibitor (FR180204) (#328007, Calbiochem) and the JNK inhibitor (SP600125) (#tlrl-sp60, InvivoGen) were used.

### Quantitative Real-Time Reverse Transcriptase-Polymerase Chain Reaction

Cells were kept overnight in starvation medium (α-MEM (11095-080, Gibco) containing 0.5% FBS (10270-106, Gibco) and 1% Penicillin/Streptomycin (15140-122, Gibco)). Further, 100 ng/ml Adiponectin (50636-M08H, Sino Biological Inc.) were added for indicated time periods: 0, 45 min, 1.5 and 3 h. Then, total RNA was isolated using the NucleoSpin^®^ RNA Kit (740955.50, MACHEREY-NAGEL). RNA concentrations were measured at 260 nm using a spectrophotometer (Nanodrop2000, Thermo Scientific). cDNA was synthesized from 1.0 μg of total RNA using the InnuSCRIPT Reverse Transcriptase kit (845-RT-6000100, Analytik Jena) and performed on CFX96^TM^ System Cycler (Bio-Rad).

The SsoAdvanced^TM^ Universal SYBR^@^ Green Supermix (1723271, Bio-Rad) was used in each reaction setup. The primers employed were: Mouse AdipoR1&2 (qMmuCID0023619, qMmuCID0010157), AP (qMmuCED0003797), BSP (qMmuCID0006396), OCN (qMmuCED0041364), OPG (qMmuCID0005205), Runx-2 (qMmuCID0005205) and F-Spondin (qMmuCED0049433) all from Bio-Rad. GAPDH (qMmuCED0027497, Bio-Rad) was used as housekeeping gene. Results were analyzed using the Bio-Rad CFX Manager 3.1 software.

### Protein Extraction and Western Blot Analysis

RIPA buffer (89901, Thermo Scientific) supplied with 3% protease inhibitor (78442, Thermo Scientific) was used for protein extraction. Protein concentrations were measured using Pierce^TM^ BCA Protein Assay Kit (23225, Thermo Scientific) on direct reading Spectrophotometer (DR/2000, HACH). Further, 20 µg protein samples were separated using 10% SDS-PAGE gel by electrophoresis and transferred to a nitrocellulose membrane (1704271, Bio-Rad). The membranes were blocked with 5% non-fat milk (T145.1, ROTH) for 1 h and incubated with the primary antibodies for Adiponectin Receptor 1 (ab70362, Abcam); Adiponectin Receptor 2 (ab77612, Abcam); ERK1/2 (MBS8241746, BIOZOL); phospho-ERK1/2 (44-680G, Thermo-Fisher); P54/P46 JNK (#9252, Cell Signaling Technology), phospho-JNK (07-175, Thermo-Fisher); P38 MAPK (#9212, Cell Signaling Technology); phospho-P38 MAPK Alpha (#4511, Cell Signaling Technology), STAT1 (AHP2527, Bio-Rad); phospho-STAT1 Tyr701 (05-1064, Thermo-Fisher); phospho-STAT1 S727 (ab109461, Abcam); STAT3 (PA1-86605, Thermo-Fisher); phospho-STAT3 S727 (OPA1-03007, Thermo-Fisher), and β-actin (ab8227, Abcam) at a concentration of 1:1,000. The secondary antibodies employed were: Polyclonal Goat Anti-Rabbit (P0448, Dako); Rabbit Anti-Goat (P0160, Dako) and Polyclonal Goat Anti-Mouse (P0447, Dako) at a concentration of 1:2000. The band signals were detected with X-ray Amersham Hyperfilm (28906836, GE Healthcare) utilizing Amersham ECL Western Blotting Detection Reagents (9838243, GE Healthcare) and visualized using OPTIMAX X-Ray Film Processor (11701-9806-3716, PROTEC GmbH).

### Immunofluorescence Staining

OCCM-30 cells were cultured overnight on sterile Falcon™ Chambered Cell Culture Slides (354108, Fisher Scientific) and further fixed with 4% paraformaldehyde (30525-89-4, Sigma-Aldrich) for 10 min at room temperature. Cells were permeabilized with 0.5% Triton™ X-100 Surfact-Amps™ Detergent Solution (28313, Thermo-Fisher) for 20 min. Then, cells were incubated in blocking buffer containing 10% goat serum, 0.3 M glycine, 1% BSA (071M8410, Sigma) and 0.1% Tween-20 (P1379, Sigma-Aldrich) for 30 min at room temperature and further incubated with primary antibodies AdipoR1 (ab70362, Abcam) (dilution 1:250) or AdipoR2 (ab77612, Abcam) (dilution 1:250) at 4°C overnight. The secondary antibodies DyLight 488 polyclonal goat anti-rabbit (ab96899, Abcam) (dilution 1:500) or donkey anti-goat Alexa Fluor 647 (ab150131, Abcam) (dilution 1:500) conjugated to fluorescein isothiocyanate were used. After washing with 1× phosphate-buffered saline (PBS) (10010023, Thermo-Fisher), samples were mounted using a fluorescent Mounting Medium with DAPI (ab104139, Abcam). Staining was analyzed using a high-resolution fluorescence microscope (Leica Microsystems, Wetzlar, Germany) and photographed.

### Cell Migration Assay

OCCM-30 cells were plated at a density of 8 × 10^3^ cells/well in 6-well plates (657160, Greiner Bio-one), in α-MEM (11095-080, Gibco) containing 10% FBS (10270-106, Gibco) and 1% Penicillin/Streptomycin (15140-122, Gibco) and cultured until confluence. Cells were preincubated for 12 h in starvation medium and wounded by scratching using a 100 μL tip. Through this, a cell-free area was created in the center of the cell layer. Afterwards, all non-adherent cells were washed with 1× PBS (10010023, Thermo-Fisher). The wounded cell monolayers were incubated in the presence and absence of different concentration of Adiponectin (50636-M08H, Sino Biological Inc.) for 24 h. Wounded-area images were taken immediately after wounding and 24 h after scratching. The wounded cell layers were photographed at ×10 magnification (Leica Microsystems, Wetzlar, Germany) and the percentages of wound closure area between cell layer borders were analyzed and calculated over time using the Image J software (National Institutes of Health and University of Wisconsin, United States).

For the MAPK inhibition experiment, OCCM-30 cells were pretreated with the P38 inhibitor SB203580 (InvivoGen), the ERK1/2 inhibitor FR180204 (Calbiochem) or the JNK inhibitor SP600125 (InvivoGen) at a concentration of 1.0 μg/mL as well as with DimethyIsulfoxide (DMSO) (D8418-50ML, Sigma-Aldrich) at 0.1% (v/v) (Control group) for 1 h before Adiponectin addition. Afterward, the pretreated OCCM-30 cells were wounded and cultivated in the presence or absence of 1.0 μg/ml Adiponectin (50636-M08H, Sino Biological Inc.).

### Cell Proliferation Assay

Cell viability and proliferation was examined using 3-(4,5-dimethylthiazol-2-yl)-5-(3-carboxymethoxyphenyl)-2-(4-sulfophenyl)-2H-tetrazolium (MTS) assay (CellTiter 96® Aqueous One Solution Cell Proliferation Assay, Promega) according to manufacturer’s instructions. Briefly, OCCM-30 cells at a passage three to five were seeded at a density of 5 × 10^3^ cells/well in a 96-well plate (655180, Greiner Bio-one). Cells were cultured in α-MEM containing 5% FBS overnight to allow adherence. Then, cells were washed twice with 1× PBS (10010023, Thermo-Fisher) and treated with various concentrations of Adiponectin (50636-M08H, Sino Biological Inc.) in α-MEM containing 0.5% FBS over a period of 24 h. To assess involvement of the MAP kinase cascade in Adiponectin-induced proliferation, cells were pretreated 1 h with the inhibitors: SB203580 (InvivoGen), FR180204 (Calbiochem) and SP600125 (InvivoGen) at a concentration of 1.0 μg/mL as well as with DMSO (D8418-50ML, Sigma-Aldrich) at 0.1% (v/v), respectively. Thereafter, 20 μL of the MTS reagent was added into each well and the cells were incubated during 2 h at 37°C in a 5% CO_2_ atmosphere. Plates were read by 490 nm using a 96-well micro-plate reader (BioTek, Winooski, VT, United States) to measure the amount of formazan by cellular reduction of MTS.

### Alizarin Red S Staining

OCCM-30 cells at passages five to seven were seeded to 6-well plates (657160, Greiner Bio-one) at a density of 3 × 10^4^ cells/well using α-MEM (11095-080, Gibco) containing 10% FBS (10270-106, Gibco) and 1% Penicillin/Streptomycin (15140-122, Gibco). Upon confluence, the culture medium was supplemented with 50 μg/ml Ascorbic Acid and 10 mM β-Glycerophosphate disodium salt hydrate with different concentrations of Adiponectin (50636-M08H, Sino Biological Inc.). Mineralization of extracellular matrix was determined on days 7 and 14 by Alizarin Red S staining. Briefly, mineralized monolayer cell cultures were washed with 1× PBS (10010023, Thermo-Fisher) three times and stained using 1% Alizarin Red S solution (A5533, Sigma-Aldrich) during 5 min at room temperature after being fixed with 70% Ethanol (64-17-5, Sigma-Aldrich) for 1 h at 4°C. Mineralized nodule formation was assessed by inverted phase contrast microscopy (Leica Microsystems, Wetzlar, Germany) using the LASV4.8 software (Leica).

To quantify the degree of calcium accumulation in the mineralized extracellular matrix, Alizarin Red S stained cultures were dissolved using 100 mM Cetylpyridinium chloride (6004-24-6, Sigma-Aldrich) for 1 h to release calcium-bound dye into the solution. The absorbance of the released dye was measured at 570 nm using a spectrophotometer (xMarkTM, Microplate Absorbance Spectrophotometer, 1681150 BioRad).

To measure the effect of MAP kinase in Adiponectin-induced cementogenesis, cells were incubated with the inhibitors: SB203580 (InvivoGen), FR180204 (Calbiochem) and SP600125 (InvivoGen) at a concentration of 1.0 μg/mL as well as with DMSO (D8418-50 ML, Sigma-Aldrich) at 0.1% (v/v) for 7 and 14 days, respectively.

### Alkaline Phosphatase Enzymatic Activity Assay

After cementogenesis induction during 14 days, OCCM-30 cells were lysed in distilled deionized water and sonicated for 15 s (SONIFIER 150, BRANSON, G. HEIHEMANA). The lysate was incubated at 37°C for 30 min with p-Nitrophenyl phosphate (p-NPP; Alkaline phosphatase Substrate, Sigma) in an alkaline phosphatase buffer solution (1.5 mM). The reaction was stopped by adding NaOH, and absorbance was read at 405 nm (xMarkTM, Microplate Absorbance Spectrophotometer, 1681150 BioRad).

### Statistical Analysis

Statistical analyses were performed using GraphPad Prism 6.0 software (GraphPad software). All values are expressed as means ± standard deviation (SD) and analyzed using one-way *t*-test for unpaired samples to determine the statistically significant differences between groups. Differences were considered statistically significant at a *p* value of < 0.05. Data distribution was analyzed using the Kolmogorov-Smirnov and the Shapiro-Wilk test and visually using QQ plots. All experiments were repeated successfully at least three times.

## Results

### Cementoblasts Express Adiponectin Receptor 1 and 2

**FIGURE 1:**
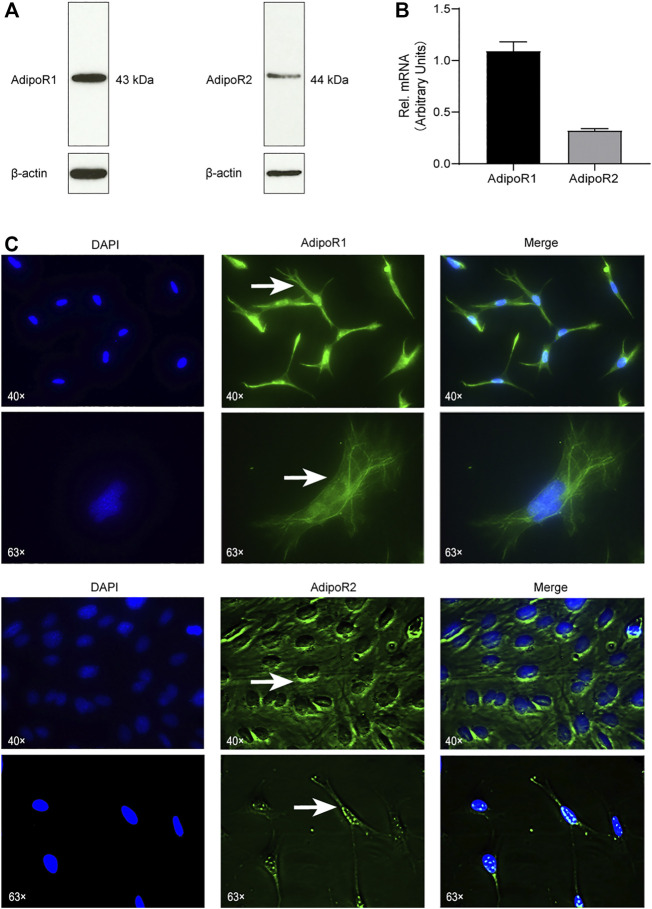
Cementoblasts express Adiponectin receptors. **(A)** The expression of Adiponectin receptors (AdipoR1 and AdipoR2) in OCCM-30 mouse cementoblasts was examined by WB. β-actin is shown as internal control protein. **(B)** RT-PCR analysis show the mRNA expression of AdipoR1 and AdipoR2. Values are expressed as means ± SD. **(C)** Adiponectin receptors in OCCM-30 cells were visualized by immunofluorescence staining (Green color). DAPI staining was used for nuclei detection. Arrows show cellular receptor localization. AdipoR1 are located in the cytoplasm, cytomembrane and nucleus, while AdipoR2 are distributed mostly around the nucleus.

First, we aimed to verify if OCCM-30 cementoblasts express Adiponectin receptors. By Western blot analysis, we could establish that AdipoR1 as well as AdipoR2 are expressed on this cell line (Figure 1A). The mRNA expression of Adiponectin receptors was also demonstrated by RT-PCR analysis (Figure 1B). Immunofluorescence staining show that AdipoR1 are mostly expressed in the cytoplasm, cytomembrane and nucleus, while AdipoR2 are expressed around the nucleus (Figure 1C).

### Adiponectin Promotes In-Vitro Cementoblast Mineralization

**FIGURE 2:**
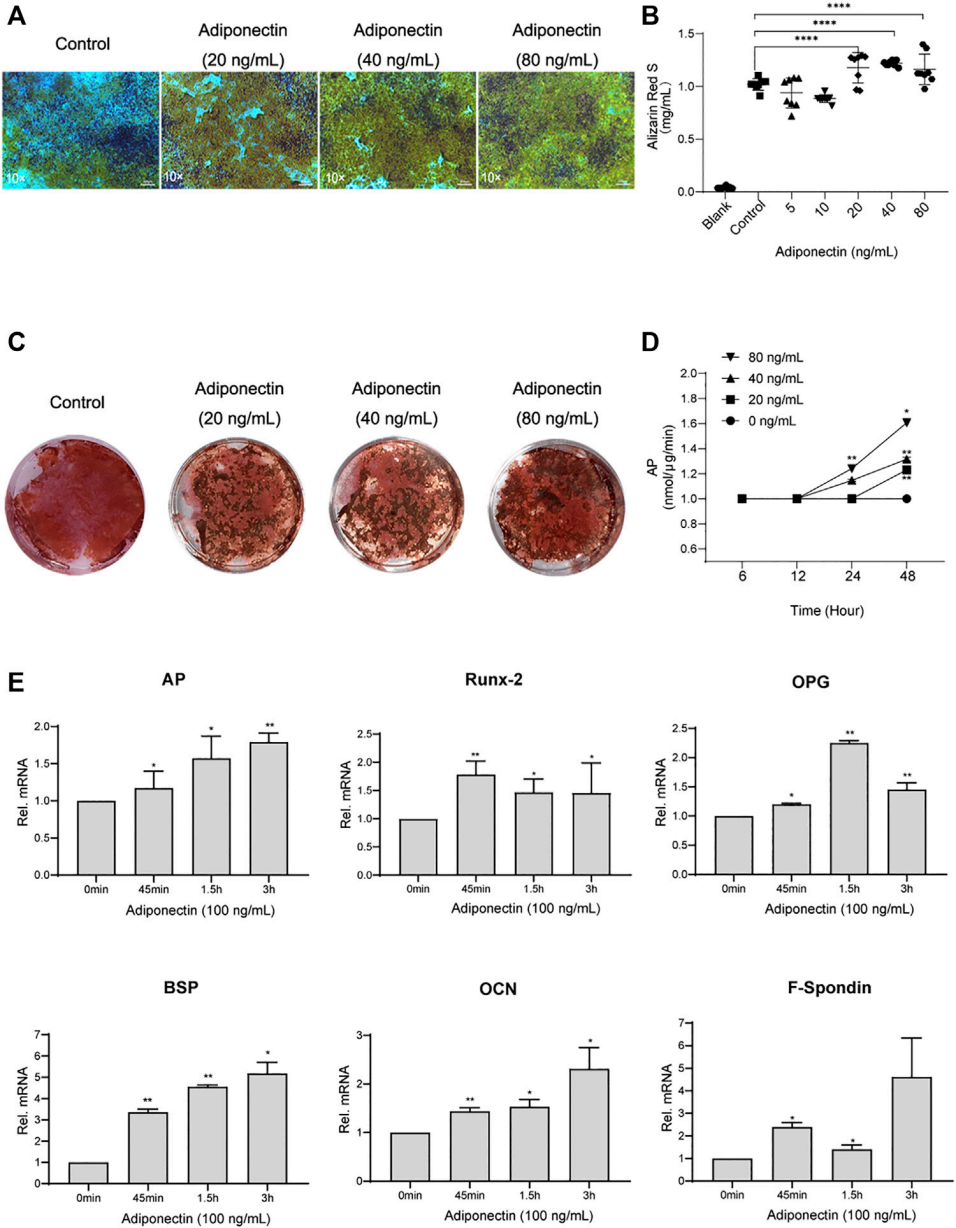
Adiponectin promotes cementogenesis *in vitro*. **(A)** Microscopic view of OCCM-30 cells after 14 days of stimulation with different concentrations of Adiponectin (Staining: Alizarin Red S). **(B,C)** Cells were cultivated with 0 (Control), 20, 40, and 80 ng/ml Adiponectin during 14 days. Mineralization grade was visualized and quantified using Alizarin Red S staining and by further dilution with Cetylpiridiumchlorid. Results are expressed as mg/ml Alizarin Red S (Mean ± SD of three independent results). **(D)** Adiponectin added to OCCM-30 cells increases the Alkaline Phosphatase (AP) enzymatic activity dose-dependently, reaching statistical significance after 24 h in comparison to the untreated group (Data are normalized to 1, *p < 0.05; **p < 0.01). **(E)** Kinetic analysis of relative mRNA expression of Alkaline Phosphatase (*AP*), Bone Sialoprotein (*BSP*), Osteoprotegerin (*OPG*), Osteocalcin (*OCN*), *Runx-2* and *F-Spondin* on cementoblasts after Adiponectin (100 ng/ml) stimulation. Data are normalized to 1. GAPDH was used as housekeeping Gene. Values are expressed as means ± SD: Ns (not significant); *p < 0.05; **p < 0.01; ***p < 0.001 and ****p < 0.0001.

Second, we analyzed the possible effect that exogenous Adiponectin exerts during cementogenesis. Alizarin Red S staining was used to visualize and quantify the biological effect of Adiponectin on OCCM-30 cell mineralization. This method revealed that Adiponectin significantly increased mineralized nodule formation in a dose-dependent manner over a period of 14 days (Figure 2A). Colorimetric analysis revealed that Adiponectin-stimulated OCCM-30 cells had higher levels of mineralized matrix production in comparison to unstimulated cells (*p* < 0.01) (Figures 2B,C).

The analysis of the Alkaline Phosphatase enzymatic activity (AP) over a period of 48 h of cells stimulated with different concentrations of Adiponectin, showed increased AP activity time and dose-dependently, reaching statistical significance (*p* < 0.01) after 24 h in the group stimulated with 80 ng/ml Adiponectin (Figure 2D).

Cells cultivated for a period of 7 days in a mineralization-inducing medium, were afterwards stimulated over a period of 3 h with Adiponectin (100 ng/ml). The kinetic analysis of the relative mRNA expression of *AP*, *Runx-2*, *BSP*, *OPG*, *OCN* and *F-Spondin* increased notably, reaching statistical significance after 45 min of stimulation with Adiponectin (100 ng/ml) in comparison to timepoint 0 min. These stimulatory effects were sustained over the entire period of 3 h (*p* < 0.05) (Figure 2E).

### Elevated Levels of Adiponectin Facilitate Cell Migration and Proliferation

**FIGURE 3:**
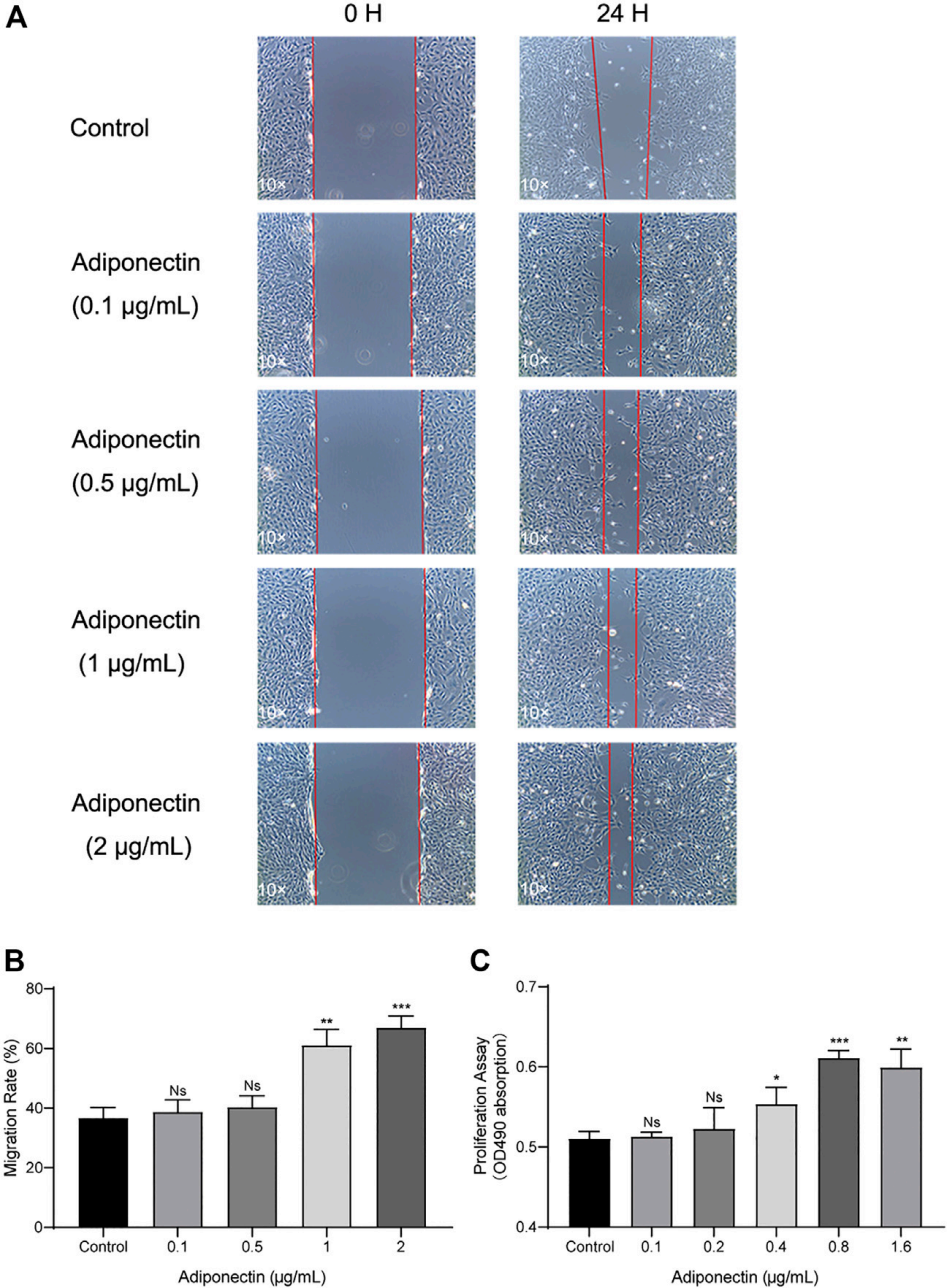
Increased migration and proliferation rates of OCCM-30 cells treated with Adiponectin. **(A,B)** Images show the migration effect that different concentrations of Adiponectin exert on OCCM-30 cells wounded monolayers at 0 and 24 h after standard scratching using a 100 µL pipet tip. The red lines indicate the wound edge at the beginning and at the end of the experiment. The migration rates were measured over a period of 24 h by ImageJ software. Data are presented as percentage of wound recovery. **(C)** The MTS assay showed that Adiponectin-treated cells during 24 h have an increased proliferation rate in comparison to untreated cells. This effect occurred dose-dependently, reaching statistical significance at a concentration of 0.4, 0.8, and 1.6 μg/ml Adiponectin. Values are expressed as means ± SD: ns (not significant); *p < 0.05; **p < 0.01; ***p < 0.001.

Next, we analyze the effect that Adiponectin exerts on cell proliferation and migration. Cells were grown to 100% confluency and then were scratched using a 100 µL pipet tip. Immediately thereafter, cells were stimulated with different concentrations of Adiponectin (0, 0.1, 0.5, 1, and 2 μg/ml) during 24 h. The cell migration ability was visualized and measured using microscopic photography (Figure 3A). The analysis of the recovered data indicates that Adiponectin at concentrations of 1 and 2 μg/ml significantly promotes wound closure (24.35 ± 2.38 and 30.3 ± 2.68%, respectively) (Figure 3B). Furthermore, we observed that cells stimulated with Adiponectin over a period of 24 h have increased mitogenic activity. The groups stimulated with 0.4, 0.8 and 1.6 μg/ml adipokine showed a significantly increased proliferation rate (*p* < 0.05) in comparison to unstimulated cells (Figure 3C).

### Adiponectin Promotes P38, ERK1/2 and JNK Phosphorylation in OCCM-30 Cells

**FIGURE 4:**
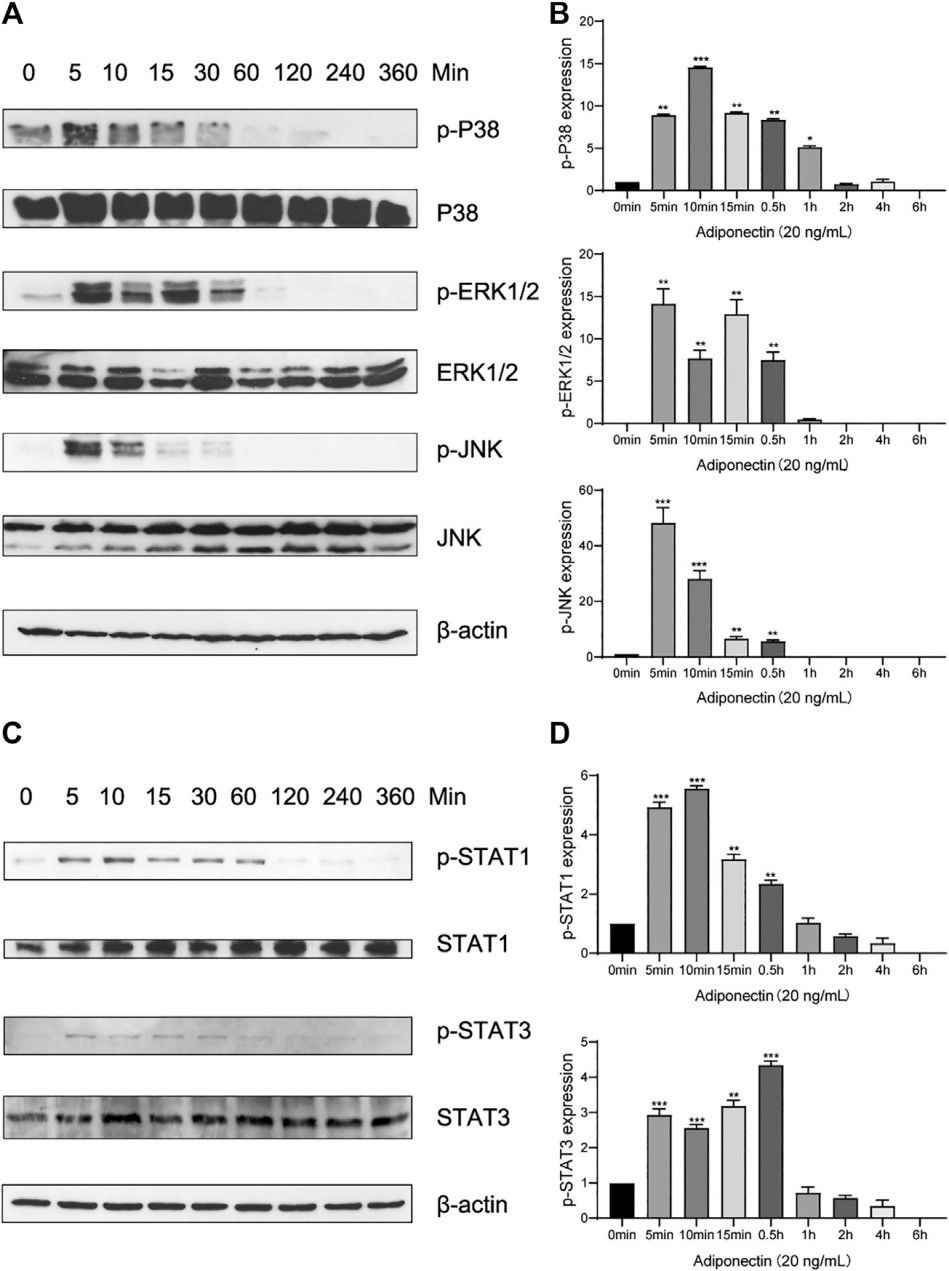
Adiponectin promotes JNK, ERK1/2 and P38 phosphorylation on OCCM-30 cells. **(A,B)** The expression of JNK (46 and 54 kDa), ERK1/2 (42 and 44 kDa) and P38 (42 kDa) expression as well as their phosphorylated forms after Adiponectin (20 ng/ml) stimulation were analyzed by Western Blots. β-actin served as a loading control. Graphics show the relative expression of p-JNK, p-ERK1/2 and p-P38 compared to cells at time point 0 min. **(C,D)** Adiponectin (20 ng/ml) promotes STAT1 (98 KDa) and STAT3 (85 KDa) phosphorylation during a period of 30 min. Graphics show the relative expression of p-STAT1 and p-STAT3 compared to cells at time point 0 min. Values are expressed as means ± SD: Ns (not significant), *p < 0.05; **p < 0.01; ***p < 0.001.

In order to elucidate if Adiponectin can activate the MAPK pathway, we performed a kinetic analysis of P38, ERK1/2 and JNK protein phosphorylation (Figure 4A).

Western blots revealed that P38 phosphorylation occurs 5 min after Adiponectin (20 ng/ml) stimulation. The phosphorylated-state of P38 was sustained over a period of 4 h, reaching a peak at time point 10 min (*p* < 0.001). The phosphorylation of ERK1/2 as well as P54/P46 JNK reached a peak after 5 min Adiponectin addition, being the WB’s bands detectable during 30 min in both cases (Figure 4B). The WB analysis revealed that cementoblasts express STAT1 and STAT3 being these proteins target of Adiponectin stimulation. The phosphorylation of STAT1 and STAT3 occurred likewise after 5 min and was detectable over a period of 30 min (Figures 4C,D).

### Blockade of MAPK Attenuates Adiponectin-Induced Cementoblast Migration and Proliferation

To evaluate whether activation of P38, ERK1/2 or JNK are essential for Adiponectin-stimulated cell migration and proliferation, OCCM-30 cementoblasts were preincubated with the pharmacological inhibitors: SB203580 (InvivoGen), FR180204 (Calbiochem) and SP600125 (InvivoGen) as well as with DimethyIsulfoxide (Sigma-Aldrich) (Control group) for 1 h and afterwards stimulated with Adiponectin.

**FIGURE 5:**
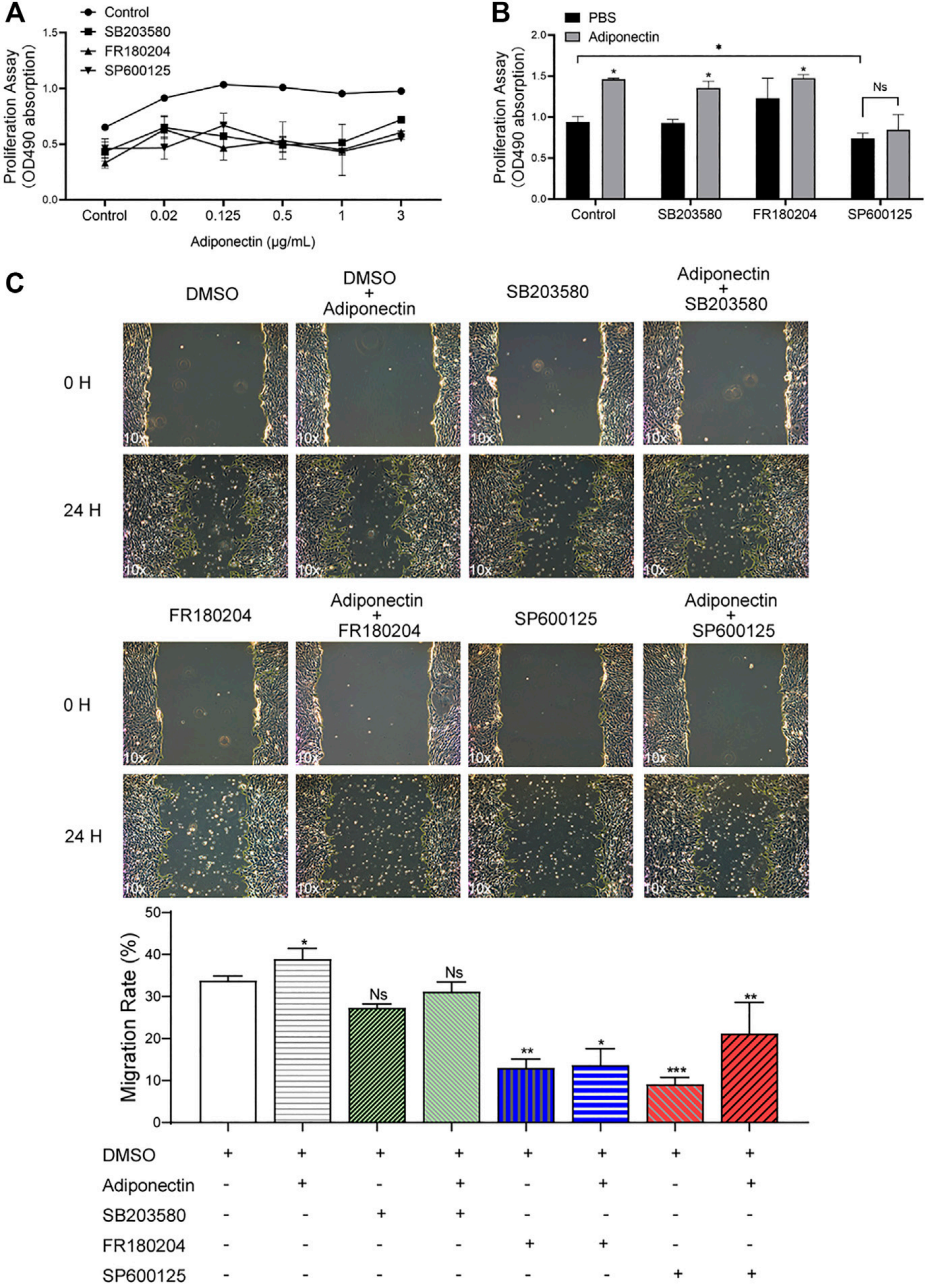
The MAP kinase pathway is involved in Adiponectin-induced migration and proliferation of OCCM-30 cementoblasts. **(A,B)** Cells were treated with the MAP kinase inhibitors: SB203580 (InvivoGen), FR180204 (Calbiochem) and SP600125 (InvivoGen) for 1 h and afterwards stimulated with Adiponectin during 24 h. As result, MAP kinase inhibition impairs cell proliferation (MTS assay). This effect was rescued after Adiponectin (100 ng/ml) stimulation. **(C)** Images of cell migration were captured prior to stimulation (0 h) and after 24 h. The recovered areas were calculated by ImageJ software comparing the scratched areas. Blockade of ERK1/2 (FR180204) and JNK (SP600125) over a period of 24 h significantly decreases cell migration whereas P38 inhibition did not significantly influence the migration rate. Values are expressed as means ± SD of three independent results: Ns (not significant), *p < 0.05; **p < 0.01; ***p < 0.001.

The MTS assay indicated that MAPK blockade reduced the cell proliferation in unstimulated and stimulated cells. However, this effect was partially counteracted by Adiponectin (100 ng/ml) (Figures 5A,B).

As shown in the pictures, the scratched areas were measured after 24 h, which is represented by a front-end yellow edge line (Figure 5C). The migratory capacity of OCCM-30 cells treated with MAPK inhibitors was examined in the presence or absence of Adiponectin (100 ng/ml). As results, the migration rate of cells was significantly attenuated after ERK1/2 as well as JNK blockade despite Adiponectin (100 ng/ml) co-stimulation, whereas this effect was not observed in the group pretreated with the P38 inhibitor (Figure 5C).

### Inhibition of MAP Kinases Alter Adiponectin-Induced Cementogenesis

**FIGURE 6:**
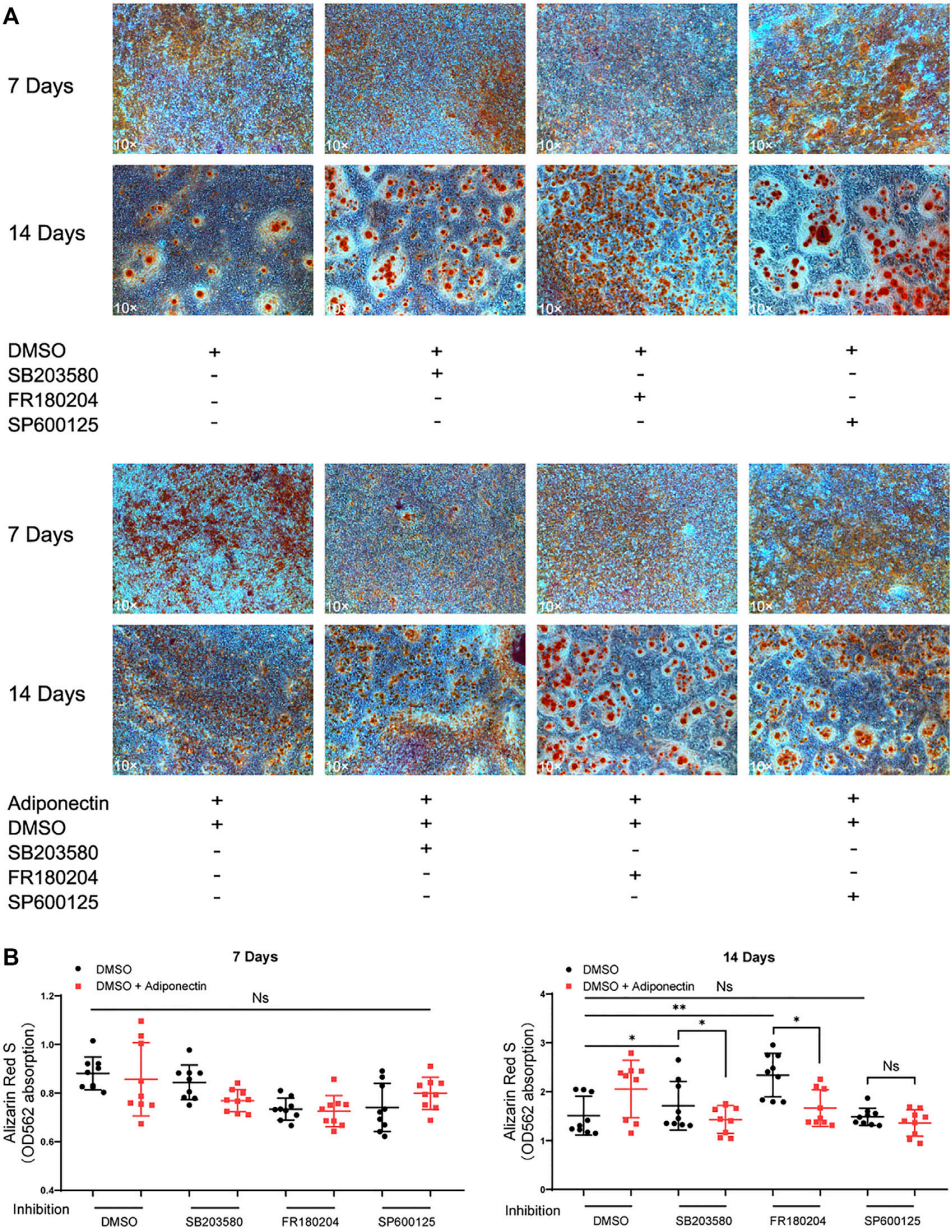
Inhibition of MAP kinases alter Adiponectin-induced cementogenesis. **(A)** Cells were treated with MAP kinase inhibitors in the presence or absence of Adiponectin (100 ng/ml) for 7 and 14 days, respectively. **(B)** Graphic shows the absorbance (OD_562_) of Alizarin Red S dilution after induction of mineralization for 7 and 14 days. Mineralization grade does not differ significantly among the groups after 7 days induction. However, after 14 days induction, the sustained blockade of P38 (SB203580) and ERK1/2 (FR180204) facilitate mineralized nodule formation, effect that was partially counteracted by Adiponectin. This effect was not observed in the groups cultivated with the JNK inhibitor (SP600125) in the presence or absence of Adiponectin. Values are expressed as means ± SD: Ns (not significant), *p < 0.05; **p < 0.01; ***p < 0.001.

The colorimetric analysis performed after dilution of Alizarin Red S staining for a period of 7 days mineralization induction, did not show significant differences among groups despite Adiponectin treatment (Figures 6A,B). Cells treated with P38 and ERK1/2 inhibitors during 14 days in the absence of Adiponectin (100 ng/ml) microscopically exhibited increased mineralization. In the presence of Adiponectin (100 ng/ml), such effect was slightly but significantly decreased (Figures 6A,B). On the contrary, the sustained blockade of JNK did not alter mineralized matrix production on OCCM-30 cells despite Adiponectin addition.

## Discussion

In the present *in vitro* study, we demonstrated that OCCM-30 cementoblasts express Adiponectin receptor 1 and 2 and that its activation facilitates cell migration, proliferation and mineralization. These effects are partially orchestrated via activation of the MAPK signaling pathway.

Clinical studies have shown that the normal salivary Adiponectin levels are around 10.92 (3.22–28.71) ng/ml and the normal serum levels are around 12.27 (8.15–14.70) μg/ml (Mamali et al., 2012). These values drastically decrease in case of obesity (Carbone et al., 2012). Reduced levels of Adiponectin as well as AdipoRs are also present in patients with severe periodontitis, a fact that suggests impaired Adiponectin function to be associated with disease severity (Saito et al., 2008; Yamaguchi et al., 2010). In our experiment, we focused on the Adiponectin effects resembling normal salivary levels (20 ng/ml) and higher Adiponectin concentrations exert *in vitro* on OCCM-30 cementoblasts.

Previous reports have shown the favorable effects Adiponectin exerts *in vitro* on the proliferation of human osteoblasts via the MAPK signaling pathway (Luo et al., 2005). In the present study, we observed over a period of 24 h, that the number of viable cementoblasts was significantly increased in presence of higher concentration of Adiponectin compared to unstimulated cells. Similar results were also obtained by Berner et al. (2004) showing that a supplementation of cell culture medium with recombinant Adiponectin enhances the proliferation of murine osteoblasts (Berner et al., 2004). In contrast to the study performed by Kanazawa et al. (2007) reporting that even low concentrations of Adiponectin stimulate the proliferation of the MC3T3-E1 osteoblast cell line (Kanazawa et al., 2007), we observed that lower concentrations of Adiponectin did not significantly stimulate cementoblast proliferation.

In our experiment we demonstrated that the expression of *Runx-2*, an essential transcription factor for osteoblast differentiation, mineralization and migration (Bosshardt, 2005; Liu and Lee, 2013) as well as for cementogenesis (Hakki et al., 2018) was strongly up-regulated in OCCM-30 cells in response to high concentrations of Adiponectin. Adiponectin-induced *Runx-2* upregulation in cementoblasts may be interpreted in the circumstances of wound healing and cementum repair and regeneration, because the regulatory role that *Runx-2* exerts in cementum formation (Kimura et al., 2018). *F-Spondin*, a cementoblast specific gene that orchestrate cementoblast differentiation (Kitagawa et al., 2012), was also up-regulated by Adiponectin. Likewise, the OCCM-30 cells presented up-regulated mRNA expression of *AP*, *BSP*, *OCN* and *OPG* after adipokine stimulation.

Alkaline phosphatase (*AP*) is essential for osteoblast (Koh et al., 1997) and cementoblasts (Hakki et al., 2018) function and is up-regulated during mineralization, thus, it is generally considered as an early marker for osteoblasts as well as for cementoblasts (Carvalho et al., 2012). It is well known that *AP* regulates the formation of cementum and is involved more in the formation of acellular than cellular cementum (Saygin et al., 2000). Here we could observe that Adiponectin strongly increases mRNA *AP* as well as AP enzymatic activity in cementoblasts.

Osteocalcin (OCN) is a non-collagenous protein that plays a regulatory role during the mineralization process (Tokiyasu et al., 2000; Thomson et al., 2003). Its expression is restricted to cells with mineralizing capacity, including osteoblasts, odontoblasts as well as cementoblasts (McKee and Nanci, 1996; Saygin et al., 2000). In our results, the up-regulation of *OCN* after Adiponectin stimulation clearly indicates that this adipokine favorably induces cementogenesis. Our *in vitro* results are consistent with previous data supporting the fact that Adiponectin can enhance the osteogenic differentiation of osteoblasts (Lee et al., 2009) as well as promote osteoblastogenesis of HPDL cells (Iwayama et al., 2012).

We could observe that Adiponectin strongly promotes mineralization in OCCM-30 cells. In concordance with these results, Wang et al. (2017) demonstrated the osteogenic capacity of Adiponectin *in vitro* using rat mesenchymal stem cells (Wang et al., 2017). Another study showed that Adiponectin enhanced the mRNA expression of *AP* and the mineralization of osteoblasts (Oshima et al., 2006). Iwayama et al. (2012) also observed that Adiponectin promotes *AP* and *Runx-2* mRNA expression as well as up-regulate the AP enzymatic activity of HPDL cells (Iwayama et al., 2012). However, in the latter study, extremely high concentrations (5–10 μg/ml) of Adiponectin were used to observe these effects (Iwayama et al., 2012). In our experiment, we used ranges of Adiponectin concentrations of among 100 ng/ml, demonstrating that murine cementoblasts require lower concentrations of this adipokine to achieve the same results. However, these concentrations still exceed the biologic concentrations of Adiponectin in saliva or in crevicular fluids (Mamali et al., 2012). In the presence of biological concentrations of Adiponectin, we could observe a moderate but not statistically significant increase of *AP*, *BSP*, *OCN* and *OPG* (Data not shown).

Our results indicate that in OCCM-30 cells, Adiponectin signals act through activation of ERK1/2, JNK as well as P38. Moreover, our present results show that activation of MAPK pathway is essential for Adiponectin-stimulated OCCM-30 proliferation and migration as these effects were partially counteracted when SB203580, FR180204 and SP600125, three highly selective inhibitors of the MAP kinase cascade, were applied. On the contrary, we observe that the sustained inhibition of P38 and ERK1/2 significantly enhances the mineralization rate of cementoblasts, effect that was partially counteracted by Adiponectin addition. These results accord with previous studies showing that long-term inhibition of the MAPK signaling pathway promotes early mineralization as well as the increase of AP activity in preosteoblastic cells (Higuchi et al., 2002).

Several *in vitro* studies have shown that Adiponectin plays a role as regulator of the MAP kinase pathways in cell homeostasis (Luo et al., 2005; Miyazaki et al., 2005; Junker et al., 2017). (Miyazaki et al., 2005) shown that Adiponectin activates the P38 and JNK pathways in myocytes (Miyazaki et al., 2005). In osteoarthritis, Adiponectin induces P38-MAPK to promote osteophyte formation (Junker et al., 2017). Interesting, Luo et al. (2005) showed that Adiponectin can activate P38 and JNK but not ERK1/2 on primary osteoblasts and that suppression of AdipoR1 abolished Adiponectin induced cell proliferation, suggesting that cell proliferation is regulated by AdipoR/JNK signaling whereas differentiation is mediated via the AdipoR/P38 cascade (Luo et al., 2005).

In a previous study, we have shown that blockade of ERK1/2 impairs leptin-induced Caspase 3 and Caspase 9 expression on OCCM-30 cells (Ruiz-Heiland et al., 2020). (Kadowaki et al., 2006) observed that Adiponectin induces osteogenesis via P38 through AdipoR1, but not AdipoR2 on C3H10/T2 cells up-regulating the expression of *Runx-2* (Kadowaki et al., 2006). However, the relation between Adiponectin and P38 stimulation may generate different outputs depending on the cell type (Kadowaki et al., 2006). For example, in calcifying vascular smooth muscle cells, the Adiponectin-P38 interactions impair osteoblastic differentiation (Luo et al., 2009). The blockade of P38 caused significant down-regulated AP activity, mineralization, and osteogenic markers such as *BSP* in odontoblast-like cells (Tang and Saito, 2018). Activity of ERKs regulates the transcription of Runx-2 during the extracellular matrices induced osteoblastogenesis of mouse pre-osteoblast cell (Xiao et al., 2002). Likewise, activation of ERKs enhances AP expression in murine osteoblastic cells (Takeuchi et al., 1997). Suzuki et al., (1999) reported that the ERK pathway promotes proliferation in osteoblastic cells, whereas the P38 MAPK pathway regulates AP activity (Suzuki et al., 1999).

## Conclusion

Our findings show that Adiponectin influences *in vitro* the migration, proliferation and cementogenesis of OCCM-30 cells partly through the MAPK signaling pathway.

## Data Availability Statement

The raw data supporting the conclusions of this article will be made available by the authors, without undue reservation, to any qualified researcher.

## Author Contributions

JY acquired and analyzed the data. JY, GR-H, and JB interpreted the data and wrote the manuscript. JY, GR-H, SR conceived, designed, and supervised the study.

## Conflict of Interest

The authors declare that the research was conducted in the absence of any commercial or financial relationships that could be construed as a potential conflict of interest.

## References

[B1] Arzate H. Zeichner-David M. Mercado-Celis G. (2015). Cementum proteins: role in cementogenesis, biomineralization, periodontium formation and regeneration. Periodontol. 2000 67 (1), 211–233. 10.1111/prd.12062 25494602

[B2] Benedix F. Westphal S. Patschke R. Granowski D. Luley C. Lippert H. (2011). Weight loss and changes in salivary ghrelin and adiponectin: comparison between sleeve gastrectomy and Roux-en-Y gastric bypass and gastric banding. Obes. Surg 21 (5), 616–624. 10.1007/s11695-011-0374-5 21331503

[B3] Berner H. S. Lyngstadaas S. P. Spahr A. Monjo M. Thommesen L. Drevon C. A. (2004). Adiponectin and its receptors are expressed in bone-forming cells. Bone 35 (4), 842–849. 10.1016/j.bone.2004.06.008 15454091

[B4] Bosshardt D. D. (2005). Are cementoblasts a subpopulation of osteoblasts or a unique phenotype? J. Dent. Res 84 (5), 390–406. 10.1177/154405910508400501 15840773

[B5] Buechler C. Wanninger J. Neumeier M. (2010). Adiponectin receptor binding proteins–recent advances in elucidating adiponectin signalling pathways. FEBS Lett 584 (20), 4280–4286. 10.1016/j.febslet.2010.09.035 20875820

[B6] Cao Z. Li J. Luo L. Li X. Liu M. Gao M. (2015). Molecular cloning and expression analysis of adiponectin and its receptors (AdipoR1 and AdipoR2) in the hypothalamus of the Huoyan goose during different stages of the egg-laying cycle. Reprod. Biol. Endocrinol 13, 87 10.1186/s12958-015-0085-1 26251033 PMC4528393

[B7] Carbone F. La Rocca C. Matarese G. (2012). Immunological functions of leptin and adiponectin. Biochimie 94 (10), 2082–2088. 10.1016/j.biochi.2012.05.018 22750129

[B8] Carvalho S. M. Oliveira A. A. Jardim C. A. Melo C. B. Gomes D. A. de Fátima Leite M. (2012). Characterization and induction of cementoblast cell proliferation by bioactive glass nanoparticles. J. Tissue Eng. Regen. Med 6 (10), 813–821. 10.1002/term.488 22499432

[B9] Caverzasio J. Manen D. (2007). Essential role of Wnt3a-mediated activation of mitogen-activated protein kinase p38 for the stimulation of alkaline phosphatase activity and matrix mineralization in C3H10T1/2 mesenchymal cells. Endocrinology 148 (11), 5323–5330. 10.1210/en.2007-0520 17717053

[B10] Chen X. Lu J. Bao J. Guo J. Shi J. Wang Y. (2013). Adiponectin: a biomarker for rheumatoid arthritis? Cytokine Growth Factor Rev 24 (1), 83–89. 10.1016/j.cytogfr.2012.07.004 22910140

[B11] D’Errico J. A. Berry J. E. Ouyang H. Strayhorn C. L. Windle J. J. Somerman M. J. (2000). Employing a transgenic animal model to obtain cementoblasts *in vitro* . J. Periodontol 71 (1), 63–72. 10.1902/jop.2000.71.1.63 10695940

[B12] de Carvalho P. M. Gavião M. B. Carpenter G. H. (2017). Altered autophagy and sympathetic innervation in salivary glands from high-fat diet mice. Arch. Oral Biol 75, 107–113. 10.1016/j.archoralbio.2016.10.033 27825677

[B13] Ding Q. Wang Z. Chen Y. (2009). Endocytosis of adiponectin receptor 1 through a clathrin- and Rab5-dependent pathway. Cell Res 19 (3), 317–327. 10.1038/cr.2008.299 18982021

[B14] Funahashi T. Nakamura T. Shimomura I. Maeda K. Kuriyama H. Takahashi M. (1999). Role of adipocytokines on the pathogenesis of atherosclerosis in visceral obesity. Intern. Med 38 (2), 202–206. 10.2169/internalmedicine.38.202 10225688

[B15] Hakki S. S. Bozkurt S. B. Türkay E. Dard M. Purali N. Götz W. (2018). Recombinant amelogenin regulates the bioactivity of mouse cementoblasts *in vitro* . Int. J. Oral Sci 10 (2), 15 10.1038/s41368-018-0010-5 29748557 PMC5966809

[B16] Haugen S. Aasarød K. M. Stunes A. K. Mosti M. P. Franzen T. Vandevska-Radunovic V. (2017). Adiponectin prevents orthodontic tooth movement in rats. Arch. Oral Biol 83, 304–311. 10.1016/j.archoralbio.2017.08.009 28866437

[B17] Higuchi C. Myoui A. Hashimoto N. Kuriyama K. Yoshioka K. Yoshikawa H. (2002). Continuous inhibition of MAPK signaling promotes the early osteoblastic differentiation and mineralization of the extracellular matrix. J. Bone Miner. Res 17 (10), 1785–1794. 10.1359/jbmr.2002.17.10.1785 12369782

[B18] Iwayama T. Yanagita M. Mori K. Sawada K. Ozasa M. Kubota M. (2012). Adiponectin regulates functions of gingival fibroblasts and periodontal ligament cells. J. Periodontal. Res 47 (5), 563–571. 10.1111/j.1600-0765.2012.01467.x 22339084

[B19] Junker S. Frommer K. W. Krumbholz G. Tsiklauri L. Gerstberger R. Rehart S. (2017). Expression of adipokines in osteoarthritis osteophytes and their effect on osteoblasts. Matrix Biol 62, 75–91. 10.1016/j.matbio.2016.11.005 27884778

[B20] Kadowaki T. Yamauchi T. Kubota N. Hara K. Ueki K. Tobe K. (2006). Adiponectin and adiponectin receptors in insulin resistance, diabetes, and the metabolic syndrome. J. Clin. Invest 116 (7), 1784–1792. 10.1172/JCI29126 16823476 PMC1483172

[B21] Kanazawa I. Yamaguchi T. Yano S. Yamauchi M. Yamamoto M. Sugimoto T. (2007). Adiponectin and AMP kinase activator stimulate proliferation, differentiation, and mineralization of osteoblastic MC3T3-E1 cells. BMC Cell Biol 8, 51 10.1186/1471-2121-8-51 18047638 PMC2214728

[B22] Katsiougiannis S. Kapsogeorgou E. K. Manoussakis M. N. Skopouli F. N. (2006). Salivary gland epithelial cells: a new source of the immunoregulatory hormone adiponectin. Arthritis Rheum 54 (7), 2295–2299. 10.1002/art.21944 16802369

[B23] Kimura A. Kunimatsu R. Yoshimi Y. Tsuka Y. Awada T. Horie K. (2018). Baicalin promotes osteogenic differentiation of human cementoblast lineage cells via the wnt/β catenin signaling pathway. Curr. Pharm. Des 24 (33), 3980–3987. 10.2174/1381612824666181116103514 30693853

[B24] Kitagawa M. Ao M. Miyauchi M. Abiko Y. Takata T. (2012). F-spondin regulates the differentiation of human cementoblast-like (HCEM) cells via BMP7 expression. Biochem. Biophys. Res. Commun 418 (2), 229–233. 10.1016/j.bbrc.2011.12.155 22244873

[B25] Koh E. T. Torabinejad M. Pitt Ford T. R. Brady K. McDonald F. (1997). Mineral trioxide aggregate stimulates a biological response in human osteoblasts. J. Biomed. Mater. Res 37 (3), 432–439. 10.1002/(sici)1097-4636(19971205)37:3<432::aid-jbm14>3.0.co;2-d 9368148

[B26] Lee H. W. Kim S. Y. Kim A. Y. Lee E. J. Choi J. Y. Kim J. B. (2009). Adiponectin stimulates osteoblast differentiation through induction of COX2 in mesenchymal progenitor cells. Stem Cells 27 (9), 2254–2262. 10.1002/stem.144 19522015

[B27] Liu T. M. Lee E. H. (2013). Transcriptional regulatory cascades in Runx2-dependent bone development. Tissue Eng. B Rev 19 (3), 254–263. 10.1089/ten.TEB.2012.0527 PMC3627420 23150948

[B28] Luo X. H. Guo L. J. Yuan L. Q. Xie H. Zhou H. D. Wu X. P. (2005). Adiponectin stimulates human osteoblasts proliferation and differentiation via the MAPK signaling pathway. Exp. Cell Res 309 (1), 99–109. 10.1016/j.yexcr.2005.05.021 15963981

[B29] Luo X. H. Zhao L. L. Yuan L. Q. Wang M. Xie H. Liao E. Y. (2009). Development of arterial calcification in adiponectin-deficient mice: adiponectin regulates arterial calcification. J. Bone Miner. Res 24 (8), 1461–1468. 10.1359/jbmr.090227 19257834

[B30] Mamali I. Roupas N. D. Armeni A. K. Theodoropoulou A. Markou K. B. Georgopoulos N. A. (2012). Measurement of salivary resistin, visfatin and adiponectin levels. Peptides 33 (1), 120–124. 10.1016/j.peptides.2011.11.007 22108712

[B31] McKee M. D. Nanci A. (1996). Osteopontin at mineralized tissue interfaces in bone, teeth, and osseointegrated implants: ultrastructural distribution and implications for mineralized tissue formation, turnover, and repair. Microsc. Res. Tech 33 (2), 141–164. 10.1002/(SICI)1097-0029(19960201)33:2<141::AID-JEMT5>3.0.CO;2-W 8845514

[B32] Miyazaki T. Bub J. D. Uzuki M. Iwamoto Y. (2005). Adiponectin activates c-Jun NH2-terminal kinase and inhibits signal transducer and activator of transcription 3. Biochem. Biophys. Res. Commun 333 (1), 79–87. 10.1016/j.bbrc.2005.05.076 15936715

[B33] Nanci A. Bosshardt D. D. (2006). Structure of periodontal tissues in health and disease. Periodontol. 2000 40, 11–28. 10.1111/j.1600-0757.2005.00141.x 16398683

[B34] Nigro E. Piombino P. Scudiero O. Monaco M. L. Schettino P. Chambery A. (2015). Evaluation of salivary adiponectin profile in obese patients. Peptides 63, 150–155. 10.1016/j.peptides.2014.11.007 25481860

[B35] Oshima K. Nampei A. Matsuda M. Iwaki M. Fukuhara A. Hashimoto J. (2006). Adiponectin increases bone mass by suppressing osteoclast and activating osteoblast. Biochem. Biophys. Res. Commun 331 (2), 520–526. 10.1016/j.bbrc.2005.03.210 15850790

[B36] Park C. H. Oh J. H. Jung H. M. Choi Y. Rahman S. U. Kim S. (2017). Effects of the incorporation of ε-aminocaproic acid/chitosan particles to fibrin on cementoblast differentiation and cementum regeneration. Acta Biomater 61, 134–143. 10.1016/j.actbio.2017.07.039 28764948

[B37] Ruiz-Heiland G. Yong J. W. von Bremen J. Ruf S. (2020). Leptin reduces *in vitro* cementoblast mineralization and survival as well as induces PGE2 release by ERK1/2 commitment. Clin. Oral Invest [Epub ahead of print]. 10.1007/s00784-020-03501-3 PMC7965856 32820432

[B38] Saito T. Yamaguchi N. Shimazaki Y. Hayashida H. Yonemoto K. Doi Y. (2008). Serum levels of resistin and adiponectin in women with periodontitis: the Hisayama study. J. Dent. Res 87 (4), 319–322. 10.1177/154405910808700416 18362311

[B39] Saygin N. E. Giannobile W. V. Somerman M. J. (2000). Molecular and cell biology of cementum. Periodontol. 2000 24, 73–98. 10.1034/j.1600-0757.2000.2240105.x 11276875

[B40] Seo B. M. Miura M. Gronthos S. Bartold P. M. Batouli S. Brahim J. (2004). Investigation of multipotent postnatal stem cells from human periodontal ligament. Lancet 364 (9429), 149–155. 10.1016/S0140-6736(04)16627-0 15246727

[B41] Suzuki A. Palmer G. Bonjour J. P. Caverzasio J. (1999). Regulation of alkaline phosphatase activity by p38 MAP kinase in response to activation of Gi protein-coupled receptors by epinephrine in osteoblast-like cells. Endocrinology 140 (7), 3177–3182. 10.1210/endo.140.7.6857 10385412

[B42] Swarbrick M. M. Havel P. J. (2008). Physiological, pharmacological, and nutritional regulation of circulating adiponectin concentrations in humans. Metab. Syndr. Relat. Disord 6 (2), 87–102. 10.1089/met.2007.0029 18510434 PMC3190268

[B43] Takeuchi Y. Suzawa M. Kikuchi T. Nishida E. Fujita T. Matsumoto T. (1997). Differentiation and transforming growth factor-β receptor down-regulation by collagen-α2β1 integrin interaction is mediated by focal adhesion kinase and its downstream signals in murine osteoblastic cells. J. Biol. Chem 272 (46), 29309–29316. 10.1074/jbc.272.46.29309 9361011

[B44] Tang J. Saito T. (2018). Elucidation on predominant pathways involved in the differentiation and mineralization of odontoblast-like cells by selective blockade of mitogen-activated protein kinases. BioMed Res. Int 2018, 2370438 10.1155/2018/2370438 29675422 PMC5838463

[B45] Thomson T. S. Berry J. E. Somerman M. J. Kirkwood K. L. (2003). Cementoblasts maintain expression of osteocalcin in the presence of mineral trioxide aggregate. J. Endod 29 (6), 407–412. 10.1097/00004770-200306000-00007 12814226

[B46] Tokiyasu Y. Takata T. Saygin E. Somerman M. (2000). Enamel factors regulate expression of genes associated with cementoblasts. J. Periodontol 71 (12), 1829–1839. 10.1902/jop.2000.71.12.1829 11156039

[B47] Wang Y. Zhang X. Shao J. Liu H. Liu X. Luo E. (2017). Adiponectin regulates BMSC osteogenic differentiation and osteogenesis through the Wnt/β-catenin pathway. Sci. Rep 7 (1), 3652 10.1038/s41598-017-03899-z 28623357 PMC5473871

[B48] Xiao G. Gopalakrishnan R. Jiang D. Reith E. Benson M. D. Franceschi R. T. (2002). Bone morphogenetic proteins, extracellular matrix, and mitogen-activated protein kinase signaling pathways are required for osteoblast-specific gene expression and differentiation in MC3T3-E1 cells. J. Bone Miner. Res 17 (1), 101–110. 10.1359/jbmr.2002.17.1.101 11771655

[B49] Yamaguchi N. Hamachi T. Kamio N. Akifusa S. Masuda K. Nakamura Y. (2010). Expression levels of adiponectin receptors and periodontitis. J. Periodontal. Res 45 (2), 296–300. 10.1111/j.1600-0765.2009.01222.x 20470261

[B50] Yamauchi N. Takazawa Y. Maeda D. Hibiya T. Tanaka M. Iwabu M. (2012). Expression levels of adiponectin receptors are decreased in human endometrial adenocarcinoma tissues. Int. J. Gynecol. Pathol 31 (4), 352–357. 10.1097/PGP.0b013e3182469583 22653349

[B51] Yamauchi T. Kamon J. Ito Y. Tsuchida A. Yokomizo T. Kita S. (2003a). Cloning of adiponectin receptors that mediate antidiabetic metabolic effects. Nature 423 (6941), 762–769. 10.1038/nature01705 12802337

[B52] Yamauchi T. Kamon J. Terauchi Y. Froguel P. Tobe K. Nagai R. (2003b). Cloning of receptors for adiponectin that mediates anti-diabetic and anti-atherogenic effects. Circulation 108 (17 Suppl.), 4–113. 10.1038/nature01705

